# Case Report: Spontaneous dissection of the renal pelvis and ureter

**DOI:** 10.3389/fmed.2026.1801321

**Published:** 2026-05-21

**Authors:** Ke Zhu, Shilei Qian, Tianyu Yang

**Affiliations:** 1 Department of Radiology, Binhai County People's Hospital, Yancheng, Jiangsu, China; 2 Department of Urology, Binhai County People's Hospital, Yancheng, Jiangsu, China

**Keywords:** computed tomography urography, hydronephrosis, obstructive uropathy, spontaneous pyeloureteral dissection, ureteral stenting

## Abstract

Spontaneous dissection of the renal pelvis and ureter is a rare urinary system disease characterized by separation between the muscularis and mucosal layers or within the muscularis of the pyeloureteral wall, with urine extravasation into the false lumen causing renal colic and hydronephrosis. We report a 30-year-old woman with no prior kidney stone history who presented with paroxysmal colic, nausea, and a 1-month history of abdominal pain that had exacerbated over 1 day; urinalysis showed multiple abnormalities. Non-contrast computed tomography (CT) revealed pyeloureteral dilation without calculi. Further computed tomography urography (CTU) demonstrated a double-lumen sign in the right renal pelvis and upper ureter, with significantly lower CT values on the dissected side suggesting urinary obstruction. The patient underwent ureteral stenting with prompt symptom relief. This case highlights that CTU reliably diagnoses spontaneous dissection of the renal pelvis and ureter, and early interventional treatment resulted in symptom relief and short-term favorable outcome in this patient, providing a valuable clinical reference for managing this rare disease.

## Introduction

Pyeloureteral dissection is a rare clinical condition of the urinary system, characterized by abnormal separation among the layers of the pyeloureteral wall. Urine extravasates into this dissected space, forming a false lumen, which can lead to symptoms such as renal colic, hydronephrosis, and urinary obstruction (1). Based on etiology, the condition can be classified into traumatic, iatrogenic, and spontaneous types (2, 3). Spontaneous dissection of the renal pelvis and ureter, in particular, is often misdiagnosed as more common disorders such as renal calculi, ureteral obstruction, or acute pyelonephritis due to the lack of clear predisposing factors and nonspecific clinical presentation.

Histologically, the pyeloureteral wall consists of the mucosa, muscularis, and adventitia, which are normally closely apposed. Ureteral peristalsis facilitates urine transport. When structural integrity is compromised by various factors, urine can infiltrate between layers under pressure, propagating the dissection and creating true and false lumens-a pathology analogous to aortic dissection. The etiology of spontaneous dissection of the renal pelvis and ureter remains incompletely understood but may involve congenital wall defects, chronic inflammatory injury, abnormally elevated intraluminal pressure, or local vascular insufficiency. Given its rarity, clinical experience in diagnosis and management is limited, and standardized diagnostic and therapeutic protocols are lacking. Therefore, timely documentation and analysis of cases are essential to refine diagnostic approaches and therapeutic strategies.

Imaging plays a central role in confirming pyeloureteral dissection. Among available modalities, CTU is the preferred diagnostic tool due to its high spatial resolution, allowing direct visualization of the double-lumen morphology, extent of involvement, and degree of obstruction. Treatment should be individualized based on symptom severity, obstruction status, and renal function. Ureteral stenting, which is minimally invasive and effective, has emerged as a key clinical intervention, effectively relieving obstruction, and improving patient prognosis.

This article presents a retrospective analysis of the clinical data, imaging findings, and treatment outcomes of one patient with spontaneous dissection of the renal pelvis and ureter, supplemented by a review of the relevant literature. The aim is to discuss key aspects in the clinical management of this condition, provide guidance for clinicians, and help reduce misdiagnosis and missed diagnoses.

## Case report

A 30-year-old female presented to our outpatient clinic with foamy urine 4 months earlier. Urinalysis findings are detailed in Table 1, with abnormal results as follows: urinary microalbumin > 150 mg/L, urine protein (2+), urine leukocytes (1+), positive occult blood, white blood cell count 28.0/μL, red blood cell count 42.0/μL, and epithelial cell count 132.0/μL.

**Table 1:** Summary of the patient’s laboratory test results.

Parameter	Result	Reference range
Color	Light yellow	Light yellow-amber
Clarity	Clear	Clear
pH	5.5	5.5–7
Specific gravity (SG)	1.025	1.005–1.025
Urinary microalbumin	>150	0–100 mg/L
Urine creatinine (Cr)	17.60	2–22 mg/L
Uric acid (UA)	2.5	2.5–10 mmol/L
Vitamin C (Vit C)	Negative	Negative
Ketone bodies (Ket)	Negative	Negative
Urine glucose (Glu)	Negative	Negative
Urobilinogen (Uro)	Normal	Negative
Bilirubin (Bil)	Negative	Negative
Leukocytes (Leu)	1+	Negative
Urine protein (Pro)	2+	Negative
Nitrite (Nit)	Negative	Negative
Occult blood (BLD)	+	Negative
White blood cell count (WBC)	28.0	0–15.7 /μL
Red blood cell count (RBC)	42.0	0–13.9 /μL
Epithelial cell count	132.0	0–8.9 /μL
Small round epithelial cells (SRC)	0.00	0–0 /μL
Bacteria (BACT)	36.0	0–100 /μL
Yeast (YLC)	15.0	0–0 /μL
Mucus Threads (MUSC)	5.0	0–0 /μL
Transparent casts	1	0–1 /LP
Granular casts	0	0–1 /LP
Calcium oxalate crystals	0	0–30
Uric acid crystals	0	0–15
Microscopic WBC	6–9	0–5 /HP
Microscopic RBC	3–7	0–3 /HP
Microscopic examination	Positive	Negative

**Figure 1:**
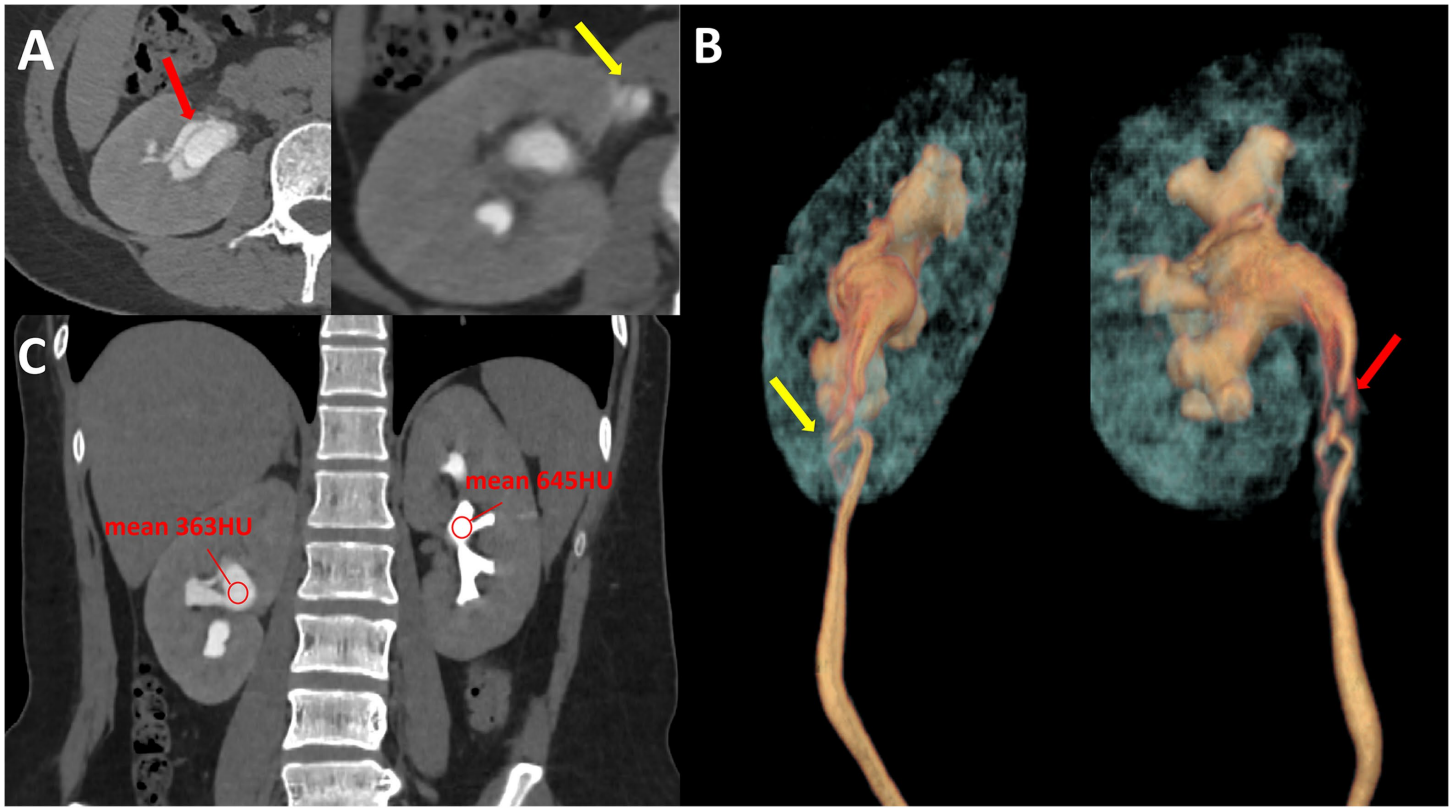
Computed tomography urography: **(A)** Axial CTU images demonstrate the presence of a double-lumen sign in the renal pelvis (red arrow) and upper ureter (yellow arrow). **(B)** VR imaging demonstrated tortuous morphology of the proximal ureter (yellow arrow) and the extent of the dissection lesion (red arrow). **(C)** During the excretory phase, the CT value of the dissected side was significantly lower than that of the contralateral healthy side.

No specific therapeutic interventions were initiated during this interval. The patient had no history of hypertension, diabetes, trauma, or prior urological interventions. She also denied any history of smoking, alcohol consumption, or long-term medication use. The patient returned to our institution in October 2025 with a one-month history of abdominal pain that had progressed acutely over the preceding 24 h. Her clinical manifestations included paroxysmal colic accompanied by nausea, with no vomiting or referred pain. Upon admission, physical examination revealed a soft abdomen with mild tenderness localized to the right upper and middle quadrants. No rebound tenderness or guarding was elicited. Costovertebral angle tenderness was absent on the right side. The remainder of the physical examination was unremarkable. A non-contrast abdominal computed tomography (CT) scan revealed right-sided pyeliectasis and ureteral wall thickening, with no evident radiopaque calculi identified. The radiology department recommended further computed tomography urography (CTU), which confirmed a double-lumen sign involving the right renal pelvis and proximal ureter (Figure 1), consistent with spontaneous dissection of the renal pelvis and ureter. Volume rendering (VR) reconstructions demonstrated a tortuous configuration of the proximal ureter, with the dissecting lesion limited to the renal pelvis and proximal ureter. During the excretory phase, the attenuation value of the dissected side was markedly lower than that of the contralateral normal side, indicative of obstructive uropathy.

**Figure 2:**
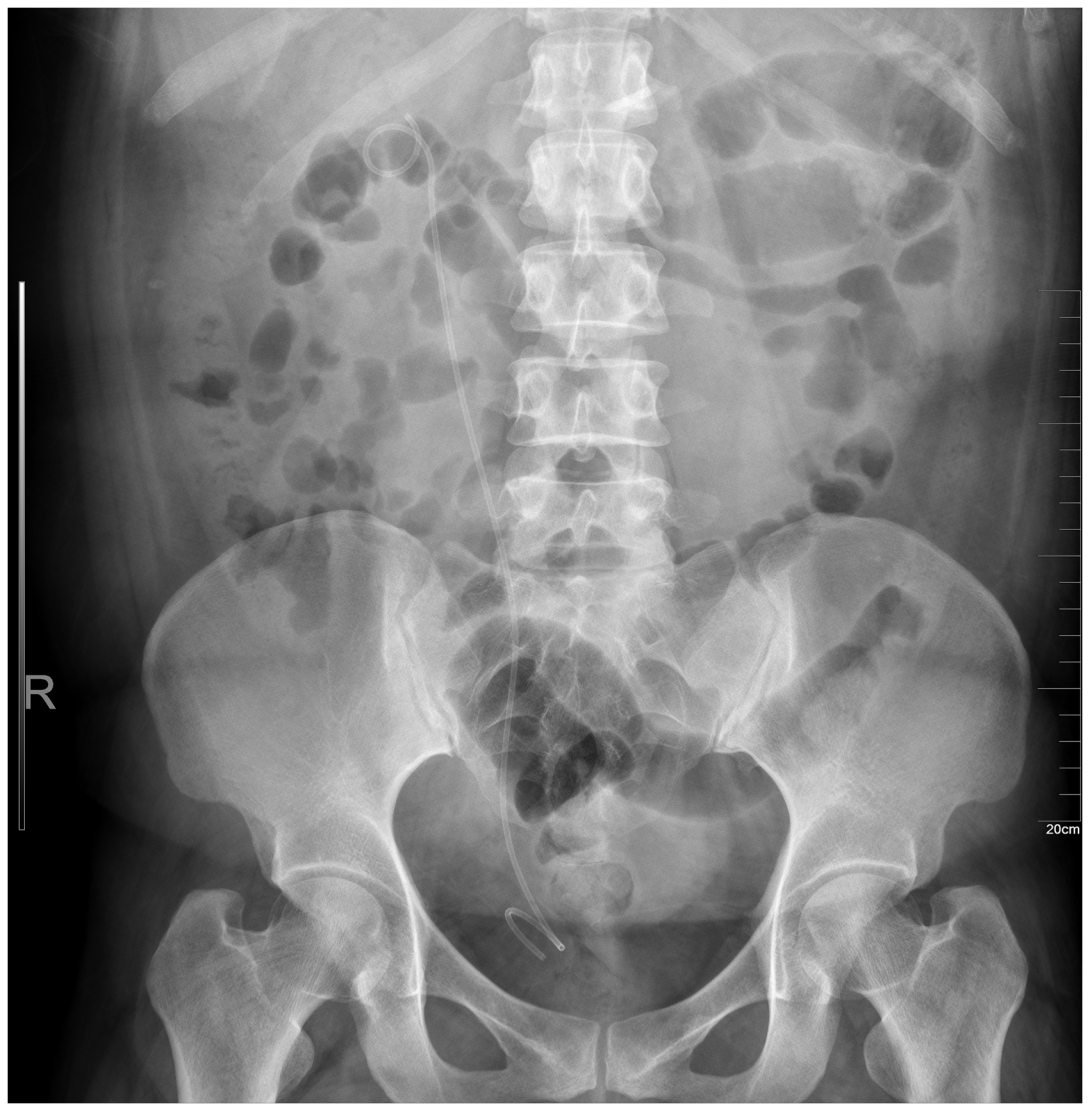
Postoperative plain abdominal radiograph demonstrating optimal positioning of the right double-J ureteral stent.

The patient was admitted for ureteral stent placement. Following successful induction of general anesthesia, the patient was positioned in the lithotomy position. A flexible 8-French ureteroscope was inserted without complication through the external urethral orifice. Both vesical ureteral orifices were identified with normal anatomical positioning and unobstructed urinary efflux. Thereafter, the ureteroscope was advanced into the right ureteral orifice, and a zebra guidewire was introduced under direct endoscopic visualization. Upon advancement of the ureteroscope over the zebra guidewire for approximately 15 centimeters proximally into the right ureter, a true-false lumen dissection was visualized. The true lumen demonstrated mild stenosis accompanied by mucosal hyperemia and edema, whereas the false lumen presented as an irregularly marginated muscular layer fissure. The zebra guidewire was carefully navigated along the true lumen; during subsequent ureteroscopic advancement, reduced ureteral wall compliance and increased passage resistance were documented. A 6-French double-J ureteral stent was then deployed over the zebra guidewire, with its proximal portion situated within the renal pelvis. The ureteroscope was gradually withdrawn, and a 16-French two-way indwelling Foley catheter was retained for bladder drainage. Postoperative abdominal plain radiography confirmed optimal positioning of the ureteral stent (Figure 2). Postoperatively, the patient’s symptoms were alleviated. She was discharged after a 3-day hospitalization. She was advised to return for imaging follow-up one month after discharge, after which the double-J stent would be removed under cystoscopy. However, due to work-related relocation to another city, the patient could not return to our hospital for the scheduled imaging follow-up. Telephone follow-up confirmed that she remained symptom-free, with no recurrence of colic or new urinary symptoms. The lack of imaging follow-up is a limitation of this case report.

## Discussion

Pyeloureteral dissection is a rare urinary tract disorder whose pathological and imaging findings resemble those of aortic dissection (4). Its etiology is not fully understood but may involve congenital wall abnormalities, chronic inflammation, elevated pressure secondary to urinary obstruction, or trauma (5). Spontaneous cases without an identifiable cause are particularly uncommon.

Clinically, patients typically present with paroxysmal renal colic as their chief complaint, often accompanied by nausea and vomiting. Some may also exhibit hematuria, fever, or other symptoms. The condition is easily mistaken for more common entities such as kidney stones or ureteral obstruction, necessitating differentiation via imaging studies. Laboratory tests often reveal elevated urinary protein, leukocytes, and erythrocytes, reflecting urinary tract inflammation and mucosal injury, which may support the clinical diagnosis (6).

Imaging is essential for confirming pyeloureteral dissection. While plain CT may show pyeloureteral dilation, it usually cannot characterize the lesion definitively. CTU, a noninvasive imaging modality, clearly visualizes the double-lumen morphology, extent of lesion involvement, and degree of urinary obstruction (7). Although CTU provides reliable morphological evidence of pyeloureteral dissection, there is currently no universally accepted diagnostic gold standard (5).

The etiology of spontaneous dissection of the renal pelvis and ureter remains incompletely understood but may involve congenital wall defects, chronic inflammatory injury, or abnormally elevated intraluminal pressure,a pathophysiology analogous to aortic dissection (5). In the present case, the patient’s urinalysis performed 4 months prior to admission revealed significant pyuria, hematuria, and proteinuria, indicating the presence of chronic urinary tract inflammation. This chronic inflammatory state may have progressively weakened the connective tissue between the muscularis and mucosa of the ureteral wall, compromising its structural integrity (8). Under normal ureteral peristaltic pressures, such weakened wall layers may be predisposed to separation, allowing urine to extravasate into the false lumen and propagate the dissection proximally into the renal pelvis, ultimately resulting in obstructive uropathy.

Pyeloureteral dissection must be distinguished from other conditions that present with renal colic and urinary tract dilation. Urolithiasis is the most common cause of acute renal colic (9), however, non-contrast CT typically reveals hyperdense calculi within the collecting system or ureter, whereas pyeloureteral dissection shows no calculi but may demonstrate a double-lumen sign on CTU. Acute pyelonephritis often presents with fever, flank pain, and pyuria (10), and contrast-enhanced CT may show abnormal renal parenchymal enhancement and haziness of the perirenal fat, but it lacks the characteristic intimal flap or false lumen seen in dissection. Incomplete duplicated collecting system is a congenital anomaly in which two ureters converge distally before entering the bladder (11). On CTU, it appears as two parallel and smoothly marginated lumina without an intimal flap, contrast extravasation, or false lumen. Cystoscopy typically reveals a single ureteral orifice on the affected side, aiding differentiation from complete duplication or ureteral dissection (12). Thus, CTU plays a crucial role in distinguishing these entities by directly visualizing the double-lumen morphology, absence of calculi, and characteristic contrast dynamics in the excretory phase.

Treatment should be individualized according to symptom severity, obstruction status, and extent of dissection. In this case, given the patient’s acute presentation with severe colic and imaging evidence of obstructive uropathy, timely decompression was indicated. Ureteral stenting was selected as a comparatively appropriate intervention due to its minimally invasive nature, ability to preserve renal function, and effectiveness in bridging the true lumen while allowing the dissected ureteral wall to heal. The patient’s symptoms were alleviated following ureteral stenting.

## Conclusion

This case highlights that CTU plays a crucial role in the definitive diagnosis of spontaneous pyeloureteral dissection. Early detection with timely ureteral stenting resulted in symptom relief and short-term favorable outcome in this patient. Although long-term follow-up was limited by logistical constraints, the diagnostic and therapeutic experience provides valuable clinical reference for managing this rare disease.

## Data Availability

The original contributions presented in the study are included in the article/supplementary material, further inquiries can be directed to the corresponding author.
